# Ethiopian mothers' experiences with micronutrient powders: Perspectives from continuing and noncontinuing users

**DOI:** 10.1111/mcn.12708

**Published:** 2019-10-17

**Authors:** Gretel H. Pelto, Alison Tumilowicz, Courtney H. Schnefke, Seifu Hagos Gebreyesus, Mélanie Hrabar, Wendy Gonzalez, Hana Yemane Wodajo, Lynnette M. Neufeld

**Affiliations:** ^1^ Division of Nutritional Sciences Cornell University Ithaca New York; ^2^ Global Alliance for Improved Nutrition Geneva Switzerland; ^3^ RTI International Research Triangle Park North Carolina USA; ^4^ Nutrition Unit, School of Public Health Addis Ababa University Addis Ababa Ethiopia; ^5^ Cape Town South Africa; ^6^ Global Alliance for Improved Nutrition Addis Ababa Ethiopia

**Keywords:** Ethiopia, evaluation, infants and young nutrition, micronutrient powder (MNP), micronutrients, qualitative methods

## Abstract

As part of a formative evaluation of a micronutrient powder (MNP) trial in Ethiopia that was organized according to a programme impact pathway model, we conducted in‐depth focused ethnographic interviews with caregivers of children between 6 and 23 months who had accepted to try “Desta,” a locally branded MNP. After stratification into two subgroups by child age, respondents were randomly selected from lists of caregivers who had received MNP from government health workers between 1 and 3 months prior to the interview date. Thirty women who were either currently giving Desta to their child (“continuing users,” *n* = 14) or had stopped feeding Desta (“noncontinuing users,” *n* = 16) were purposefully recruited from both urban and rural areas in the two different regions where the trial was conducted. Interviews were recorded, transcribed and translated, and coded for both emerging and prespecified themes. On the basis of identifiable components in the caregiver adherence process, this paper focuses exclusively on factors that facilitated and inhibited “appropriate use” and “continued use.” For “appropriate use,” defined as the caregiver preparing and child consuming MNP as directed, we identified four common themes in caregiver narratives. With respect to “continued use,” the caregiver providing and child consuming the minimum number of MNP sachets over a recommended time period, our interviews spontaneously elicited five themes. We also examined caregivers' perceptions related to problems in obtaining refills. Attention to caregivers' perspectives reflected in their narratives offers opportunities to improve MNP utilization in Ethiopia, with potential application in other social and cultural settings.

**Key messages**

- Many mothers said their child differentiated between foods with MNP and foods without MNP.
- When health workers advise mothers about side effects, it moderates the mothers' reactions when those side effects occur.
- Findings call attention to the need for support systems for caregivers to manage negative child reactions to food prepared with MNP.
- Our study highlights the need for further research on mother–child interactions around food, as well as the development of taste preferences at different ages and stages of development, particularly in resource‐constrained environments.

## 1 INTRODUCTION

In Ethiopia, modelling optimal complementary feeding diets has demonstrated that local foods cannot meet iron and zinc requirements and additional interventions are needed (Osendarp et al., 2016). Moreover, only about 7% of infants and young children 6–23 months of age consume the recommended minimum number of food groups associated with better nutrient adequacy and growth (Arimond & Ruel, 2004; Central Statistical Agency Ethiopia and ICF International, 2016; Marriott, White, Hadden, Davies, & Wallingford, 2012; World Health Organization [WHO], 2008). The Global Alliance for Improved Nutrition (GAIN) and Concern Worldwide supported the Federal Ministry of Health (FMOH) in Ethiopia for a trial to deliver micronutrient powder (MNP) through the FMOH's Health Extension Program, which deploys government‐salaried female health extension workers (HEW) to provide primary health care services in rural communities (Federal Ministry of Health: Health Extension and Education Center, 2006; Federal Ministry of Health: Health Extension and Education Center, 2007). MNP, a single‐dose packet of dry powder containing lipid‐encapsulated iron and other micronutrients that can be sprinkled onto any semisolid food (Zlotkin et al., 2005), is recommended by the WHO to address anaemia and iron deficiency in young children (WHO, 2011, 2016). From May 2016 to May 2017 in 11 *woredas* (geographic units equivalent to districts) of Amhara and Tigray Regions in Ethiopia, HEW integrated the delivery of MNP sachets and recommendations in their routine services, which include growth monitoring, cooking demonstrations, health and nutrition interpersonal communication (e.g., counselling), and immunizations.

**Figure 1:**
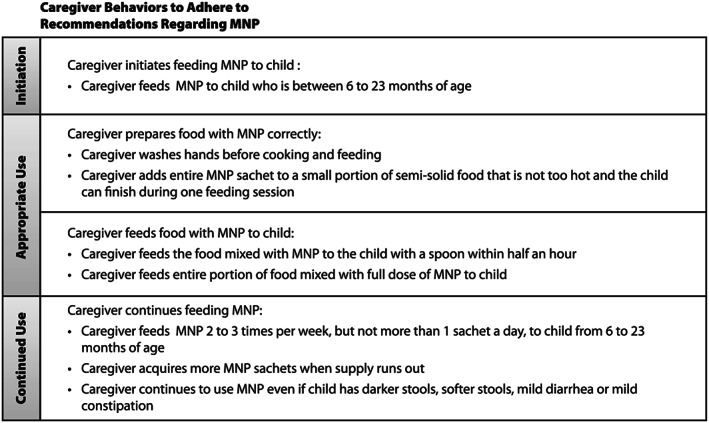
Programme impact pathway depicting household delivery system of micronutrient powder. Caregiver behaviours based on key messages communicated as part of Ethiopia programme. Adapted from (Tumilowicz et al., 2017)

To inform the scale‐up of MNP delivery through the FMOH's Health Extension Program, a theory‐driven formative process evaluation was conducted using a mixed‐method approach (Rawat et al., 2013). Both survey methods and focused ethnographic study (FES) methods were employed to describe and understand how the intervention worked, with the goal of determining what aspects of programme delivery and adherence went well; identifying where problems occurred, the magnitude, and causes of problems; and providing knowledge and insights to support potential for impact and inform expansion of the intervention. For MNP interventions, adherence to MNP recommendations involves three main elements: initiation (caregiver determination to feed MNP and starting to do so), appropriate use (caregiver preparing and child consuming MNP as directed), and continued use (caregiver providing and child consuming the minimum number of MNP sachets over a recommended time period). Figure 1 identifies caregiver behaviours for the three elements, based on the specific instructions provided in the Ethiopia programme.

This paper presents key findings from the FES that was conducted with caregivers who participated in the programme. The purposes of this paper are (a) to contribute to the growing body of empirical data on household responses to MNP interventions, with an emphasis on caregivers' perspectives, and (b) to contribute to generating theories in implementation science of nutrition interventions, particularly with respect to interventions that are directed to vulnerable infants and children. In Section 3, we present the main themes that emerged from our ethnographic analysis of two components of adherence to MNP recommendations, specifically (a) appropriate use and (b) continued use. We focus on these two aspects of adherence because the cross‐sectional survey results of programme outcomes showed that virtually all caregivers who received the MNP initiated feeding it to their children (98%; Tumilowicz et al., 2019). However, a much smaller proportion of caregivers who initiated use of MNP reported using it within 14 days prior to the survey (49%), making that one of the largest challenges for potential impact of the intervention.

## 2 METHODS

### 2.1 MNP integration into FMOH Health Extension Program

The study, which took place in Amhara and Tigray Regions, was designed to assess both programme delivery and household delivery of MNP, building on a model that was initially developed by Alive & Thrive (A&T) and implemented through the FMOH's Health Extension Program (Kim et al., 2015; Kim et al., 2016). The locally branded MNP, “Desta” (the Amharic and Tigrinya word for “joy, happiness”), together with behaviour change communication (BCC) activities, was delivered to approximately 71,000 children 6–23 months in six *woredas* in Amhara Region (Kobo, Gubalafto, Delanta, Dessie Zuria, Kalu, and Bati) and five *woredas* in Tigray Region (Saesi Tsaedaemba, Mereb Lehe, Werie Lehe, Gulomahda, and Ahferom). Building on the A&T foundation, GAIN created guidelines, a training manual, job aids, and materials for caregivers, which included information on the composition and purpose of Desta, instruction on food preparation with Desta, and contraindications and potential side effects of Desta. Implementation materials also included procedures for performance monitoring, supply chain management, and delivery of BCC activities protocols. GAIN trained “master trainers” from CWW and FMOH in October 2015, who subsequently trained CWW staff, FMOH health workers, and HEW from April to July 2016.

HEW or health centre staff provided a box containing 30 Desta sachets every 2 months to caregivers at the health post, health centre, or during home visits. At the initial distribution, caregivers were provided with a “Desta child card” that could be used to keep track of distribution dates. The card included appointment dates for caregivers to return to receive a new box of Desta, which was scheduled 2 months after the date of the current distribution. HEW and health centre staff were also trained to communicate the key messages shown in Table 1 during routine counselling sessions and cooking demonstrations; both of which formed part of the BCC approach.

**Table 1:** Key messages regarding Desta that health extension workers were trained to provide to caregivers

Topic	Key message
Composition	One sachet of Desta provides the recommended daily amount of 15 important vitamins and minerals for young children 6–23 months old. Desta is not medication.
Benefits	Improves child's health and development. Improves child's appetite. Improves the nutrition quality of child's food. Prevents anaemia and micronutrient deficiencies.
Administration regimen	Give Desta every other day. The child needs to have regular consumption to benefit from Desta. One box contains 30 sachets. Give all of them to the child within 2 months. Then you will receive a new box until your child reaches the age of 2 years.
Preparation	Wash your and your child's hands with soap and clean water before cooking and feeding child. Only add Desta to soft or mashed food. Do not mix Desta with liquids. Examples of the types of foods that could be mixed with Desta include the following: ○ Thick porridge ○ Mashed vegetables, for example, potato, carrot, and kale ○ *Shiro* (stew with chickpea flour‐based paste or lentil based thick paste with injera [flat bread with a slightly spongy texture made of *teff*, barley, or maize flour]) ○ Mashed banana, avocado, mango, or papaya Do not add Desta during cooking or to hot food. Mix Desta after food has cooled. Desta should be mixed with a small amount of food that the child can finish during one feeding session. Pour the whole sachet of Desta into the child's food and mix well. Content of one whole sachet should be mixed with one meal for one child. It is not recommended to split one sachet into portions given over several meals. Feed child immediately or within 30 min of mixing Desta with food. Do not share the food mixed with Desta with other household members. Do not feed more than one sachet of Desta per day. Store Desta in a clean, cool, and dry place.
Side effects	There are no major side effects associated with Desta. In the first few days of taking Desta, the child's stool may be darker than usual. This is completely normal and caused by the iron contained in Desta. Continued use of Desta is advised. During the first days of taking Desta, the child may have softer stools, mild diarrhoea, or a mild form of constipation, which usually passes in a period of 4–5 days. This does not happen to all children. This is also normal, and it should not be cause for concern. If diarrhoea is severe, bloody, or with mucous, take the child to the health facility for care as you would have without concurrent use of Desta.
Contraindications	Children suffering from severe acute malnutrition should not receive Desta. Desta is unsuitable for treatment of severe acute malnutrition and children should receive the usual ready‐to‐use therapeutic foods (RUTF) instead. Desta can be used safely after a child is rehabilitated. Children suffering from malaria should temporarily stop consuming Desta. Once malaria treatment has been completed, children can continue to consume Desta.

### 2.2 Study sites

Tigray Region has a population of almost 5 million people, of which 20% are urban (Central Statistical Agency of Ethiopia, 2007). Average household size is 4.4 persons who are predominantly (96%) Tigrinya‐speaking Tigray ethnic group. Agriculture is the main occupation. Amhara Region has a population of over 17 million people, of which 12% are urban (Central Statistical Agency of Ethiopia, 2007). Average household size is 4.3, and 92% are from the Semitic‐speaking Amhara ethnic group. Agriculture is also the main occupation. Interviews in Tigray were conducted in six rural *woredas* and one urban area, and in Amhara, they were conducted in two rural *woredas* and one urban area.

### 2.3 Design and sampling

FES is a mixed‐method research approach that is designed to answer specific sets of questions from the perspective of the target population that are important for various decision‐making activities, including improving programme delivery and utilization of interventions (Pelto & Armar‐Klemesu, 2015; Pelto, Armar‐Klemesu, Siekmann, & Schofield, 2013; Pelto, Dufour, & Goodman, 2013). FES draws from several qualitative and quantitative ethnographic methods. In this study, we used in‐depth interviews with open‐ended, guided questions, which were administered with extensive probing to expand and interpret the initial responses. Our interview protocol contained four modules that covered a range of issues including demographic and socio‐economic status (SES) characteristics; experiences receiving Desta and participating in BCC activities; motivation for initiating and continuing to feed Desta; motivation for discontinuing Desta; preparation of food with Desta; perceived changes in food mixed with Desta; perceived reactions of child when fed food with Desta, initially and over time; caregiver responses when a child refuses to eat food mixed with Desta; beliefs about how to keep children healthy; beliefs about the benefits of Desta; perceived changes as a results of the child consuming Desta; and how programme delivery of Desta could be improved (Schnefke et al., 2019). Interviews were conducted in mothers' homes from November to December 2016 and averaged 2–3 hr.

Respondents were randomly selected from lists of mothers of children 6–23 months of age who had been registered by HEW as receiving Desta within 1–3 months prior to the interview date. The sample design required filling respondent categories based on subgroups of the 6‐ to 23‐month age range. We aimed to interview six mothers of children 6–11 months of age and nine mothers of children 12–23 months of age in each region. We also sought to interview caregivers who reported currently using Desta (referred in this paper as “continuing users”) and women who had initiated using Desta but had stopped using it before the point at which they were contacted for an interview (referred to as “noncontinuing users”).

Table 2 presents a summary of selected sociodemographic characteristics of the 30 respondents. All interviewed caregivers except for one who was the grandmother were the biological mother of the index child. Therefore, in the rest of this paper (and in the title), we refer to the respondents as mothers. The sample was relatively homogenous in important sociodemographic characteristics, such as education, occupation, and household composition. Most mothers said they were engaged in farming or in a combination of household responsibilities and farming, and some reported engaging in petty trade. Depending on the season, mothers spend many hours each week away from the house. The majority of respondents lived in nuclear families, whereas the rest lived in extended families of varying composition, including fathers‐in‐law, mothers‐in‐law, brothers‐in‐law, older generation mothers, fathers, and sisters, or grandchildren.

**Table 2:** Selected sociodemographic characteristics of respondents and their children

		Amhara (*n* = 15)	Tigray (*n* = 15)
Household and caregiver characteristics			
Age of respondent (year)		25.9 ± 4.5[Link] (19,35)	27.9 ± 4.6[Link] (20,37)
Living with father of index child (*n*, yes)		15	13
Is biological mother of index child (*n*, yes)		14^b^	15
Schooling of caregiver (*n*)	Illiterate	5	4
	Primary education (Grades 1–8)	7	9
	Secondary education (Grades 9–12)	3	2
Occupation of caregiver in addition to household duties (*n*)	Farming	9	5
	Petty trading	2	3
	None	4	6
Household size		4.3 ± 1.0[Link] (3,6)	4.6 ± 2.1[Link] (2,9)
Child characteristics			
Age of index child (*n*, month)	6–8	2	2
	9–11	4	3
	12–23	9	8
Sex of index child (*n*)	Male	6	10
	Female	9	5
Breastfed (*n*, yes)		15	15
Currently being fed food with Desta (*n*, yes)		8	6

Mean and standard deviation; range in parentheses.

b One caregiver was the grandmother of the child.

The study protocol was approved by the Amhara National Regional State Health Bureau Ethics Review Committee and the Government of the National Regional State of Tigray, Bureau of Health Ethics Review Committee. GAIN requested and received permission from the FMOH to undertake the evaluation. The FMOH communicated its permission for the evaluation to the regional health bureaus, zonal health offices, and *woreda* health offices. After the nature of the study was fully explained to the participants in their local languages, written informed consent was obtained from all respondents (for nonliterate respondents, a thumbprint was obtained in the presence of witnesses). There were no refusals. Respondents were informed that (a) they had right to withdraw from the study at any time, (b) individual study results would be treated confidentially, and (c) their responses would not affect any food or nonfood distributions.

### 2.4 Data analysis

Thematic analysis of text (using the transcribed and translated recordings of the interviews) followed basic principles of qualitative text analysis (Miles, Huberman, & Saldaña, 2014). We used QSR International's NVivo Version 11 qualitative data analysis software to organize data and facilitate coding (QSR International, 2002). Data analysis of the caregiver interviews was conducted as a team through sharing NVivo project files, meetings, and memos summarizing actions taken regarding coding and emerging themes. First, double coding was completed on a sample of transcripts. From those initial transcripts, the team developed a common coding framework and assessed intercoder reliability. Coding then continued for the remaining transcripts with on‐going input from members of the study team. During weekly study team meetings over the course of 3 months, we adjusted the coding framework, identified emerging themes, and created matrices of quotations that were used to compare themes across transcripts.

## 3 RESULTS

In this paper, we are concerned with two key components of caregiver behaviour with respect to MNP adherence: “appropriate use,” the caregiver preparing and child consuming MNP as directed, and “continued use,” the caregiver providing and child consuming the 30 MNP sachets within a 2‐month period. We begin with “appropriate use,” in which we identified and coded four themes in the mothers' narratives. These results are followed by the presentation of five themes pertaining to “continued use” that emerged spontaneously in the caregivers' narratives. The last part of the results section presents the findings from narratives that were obtained from a general question near the end of the interview, which was phrased as “Could you tell me how you collect a new supply of Desta?”

### 3.1 Appropriate use

Appropriate use refers to mothers preparing and children consuming MNP as directed in programme guidelines that are intended to be communicated by HEW. Mothers overwhelmingly reported following recommended preparation instructions, and these instructions were followed by both continuing users and noncontinuing users (Table 3). (It should be noted that direct observation of transmission of these messages was not done.) Porridge and mashed *fitfit* (consisting of *injera* [flat bread with a slightly spongy texture made of teff, barley, or maize flour] with *shiro* [stew with chickpea flour‐based paste or lentil‐based thick paste]) were the most commonly reported foods mothers mixed Desta with for children 6–11 months and 12–23 months old.

#### 3.1.1 Theme 1. Inter‐personal communication by the HEW and cooking demonstrations played a central role in mothers' reports pertaining to knowledge and confidence to prepare and feed food with Desta

Mothers reported that HEW verbal instructions and cooking demonstrations showed them how easy it is to add Desta to foods and gave them confidence that they could use this new product correctly:

The HEW taught me about Desta, about its contents, benefit and on what food to be added. The cooking demonstration on how to use it was clear for me, those things made me decide to use Desta for my child.—Noncontinuing user from Tigray

I was wondering how this thing is going to be given to the child. But then they taught us how, so I took it and went home.—Continuing user from Amhara

**Table 3:** Instructions that mothers reported following regarding preparation and feeding of food mixed with Desta (*n* = 30, including 14 continuing users and 16 noncontinuing users)

	Continuing user (*n*, yes)	Noncontinuing user (*n*, yes)
Wash your and your child's hands with soap and clean water before cooking and feeding child	9	6
Only add Desta to soft or mashed food. Do not mix Desta with liquids	13	15
Do not add Desta during cooking or to hot food. Mix Desta after food has cooled	11	14
Desta should be mixed with a small amount of food that the child can finish during one feeding session	12	14
Add entire content of sachet at one meal. Do not split sachet over several meals	13	16
Feed child immediately or within 30 min of mixing Desta with food	13	16
Do not share food mixed with Desta with other household members	11	14
Do not feed more than one sachet per day	13	14
Give Desta every other day	8	7
Give 30 sachets of Desta to the child within 2 months	3	3
Store Desta in a clean, cool, and dry place	1	2

One third of the mothers were nonliterate, and several stated that this kept them from being able to use the materials, written in Tigrinya and Amharic, that the HEW provided. Some mothers overcame this by having older children read the materials to them, but others emphasized the importance of HEW's interpersonal, verbal communication:

Firfir and porridge, these are the only foods that I add Desta to. They told me that I should not add Desta in atmit [gruel] so I am doing exactly what they told me. I am not literate so it is difficult for me to add it on other foods, but when they taught us they told us to put it in porridge and firfir, so it's hard to put it in something they haven't told us.—Continuing user from Amhara

#### 3.1.2 Theme 2. Mothers reported changes in organoleptic characteristics of food mixed with Desta and linked these to preparation techniques

In theory, MNP is a bland powder that can be added to any food, provided it is not a liquid and that the food is not so hot as to melt the lipid layer that protects the iron from interacting with the food. However, most of the respondents (*n* = 24) reported organoleptic changes, including alterations in colour (*n* = 16), taste (*n* = 12), smell (*n* = 10), and/or texture (*n* = 7). Eleven mothers (seven continuing users and four noncontinuing users) noted some of these organoleptic differences only occurred if certain factors were at play. For example, some respondents said there would not be any change if Desta was “well mixed into the food.” Others described changes if Desta was mixed into food that was still being cooked or hot or if the food “stayed too long.” Mothers' awareness of these changes, and the accompanying preparation explanation, was attributed by them to their attendance at a cooking demonstration, by experiencing the change first‐hand, or from verbal education they received from the HEW:

If it is added on hot food it will bring change. They showed us what type of change it will bring if it is added to hot food. They prepared porridge and showed us what will happen if we add it to hot food, so I will not add on hot food. I will add it after it is cooled, so it has no change.—Noncontinuing user from Amhara

Sometimes he did not consume the food completely. This happened when he got sick because he has a common cold. Also, if I did not properly prepare it because of many reasons, such as if I am hurrying to do other jobs. Meanwhile, I was not perfect on how to prepare Desta, especially at the very beginning of Desta feeding. I did not know how to prepare Desta and as a result, he repeatedly refused to eat Desta, but later on, I got advice from the HEW and I attended the demonstration on the distribution day. Now I am an expert on how to prepare Desta and as a result my child is comfortable with Desta. I can assure you that if Desta is prepared well, it does not have a difference with the other foods that do not contain Desta. Our children may refuse Desta if we do not prepare it correctly.—Continuing user from Tigray

#### 3.1.3 Theme 3. Mothers noted children's initial rejection of food with Desta, often followed by acceptance with continued exposure

Twelve mothers said that their child initially did not seem to like the food mixed with Desta (e.g., made a “bitter face” or a face “as if she ate something sour”) or refused to eat (e.g., turn their head away from the food) but eventually “got used to it” or accepted it. A few mothers offered (or were prompted to provide) an explanation as to why they thought their child initially did not seem to like it. One said because her child's first experience with Desta was also his first experience with complementary foods, so perhaps, “[his unpleasant expression] could be because of his first‐time exposure to food in general.” Another mother thought her child initially did not like it simply “because it's a new thing.” One suggested that her child was not taking any food well when she first started feeding Desta, but the Desta improved her child's appetite so that the child was now taking all foods, including those mixed with Desta.

#### 3.1.4 Theme 4. Mothers reported various strategies and techniques to encourage children to eat food with Desta

Almost all mothers (*n* = 26, thirteen continuing users and 13 noncontinuing users) described feeding techniques or problem‐solving strategies they used to encourage their child to eat food with Desta. Some of these strategies were employed proactively, and some were reactive. Preparing Desta with a variety of foods, or trying Desta with different foods if a mother perceived her child did not like it with certain foods, was the most commonly reported strategy (*n* = 11). This was followed by descriptions of playing with or “fooling” the child (*n* = 8) and then by force‐feeding (*n* = 5) or feeding Desta at a time the mother knew her child would be hungry (*n* = 4). Other less common strategies included waiting a bit of time and then trying again, either hand‐feeding or letting the child self‐feed, and making the food “tastier.” Two continuing users also described mixing in only half of a Desta sachet into food until their children got “used to it,” perhaps for the first week or so. Although this practice is not in line with the instruction that the mother should mix the entire contents of the sachet into the food, the mothers' technique may have facilitated the acceptance of foods with Desta by these two children. The most commonly reported strategy for ensuring children ate all the food was to mix the Desta in with the amount of food the mother thought her child could finish eating at one time (*n* = 12).

### 3.2 Continued use

The final step in the adherence process is the continued use and feeding of MNP. Themes 5 through 9 relate to continued feeding versus stopping based on the narratives of both continuing and noncontinuing users. Continuing users reported feeding Desta between 1 and 9 months before the interview. As shown in Table 4, nine of the 16 noncontinuing users reported feeding Desta to their children for 3 months or longer before discontinuing its use. The most frequently cited reasons for discontinuing Desta were child refused food with Desta (*n* = 9), no perceived benefit (*n* = 4), trouble obtaining refill (*n* = 4), and diarrhoea (*n* = 3). Mothers often mentioned several challenges rather than just one cause for discontinuing use of Desta.

#### 3.2.1 Theme 5. Perceptions of positive changes in their children are reported commonly by caregivers who continued to use Desta

Closely tied to learning about Desta's benefits for children, which emerged strongly in mothers' decisions narratives about why they decided to try preparing and feeding foods with Desta to their child, is their articulation of observing benefits when they used it. Two thirds of mothers (*n* = 20, twelve continuing users and eight noncontinuing users) reported perceiving a variety of positive changes in their child after starting Desta (Table 5). The following quotations from continuing users in Amhara describe various aspects of this theme:

**Table 4:** Noncontinuing user reasons for not feeding Desta at the time of the interview

Region	Child age at initial use (month)	Estimated time fed Desta (month)	Reason for discontinuing use	Quote from narrative
1. Amhara	6	9	Child refused food with Desta	“Desta is so useful for children, but she refused to take it. It will help them to be protected from diarrhoea and abdominal discomfort … I tried all my best since it is very essential for her … I wanted to feed her … but she refused. I tried but she refused.”
2. Amhara	16	3	Perceived Desta nauseated child; child refused food with Desta	“She vomited when she tasted food with Desta. It nauseated her severely. But she eats very well when I give her other foods … she eats well when I give her any other thing. I decided the problem is with Desta and stopped giving her. I did not force her.”
3. Amhara	16	6	No perceived benefit; child refused food with Desta; perceived child lost appetite because of Desta	“I think maybe it didn't work for him. It makes him lose appetite and I thought maybe it is not tasty for him, maybe that is why I have not seen change. This is just my opinion.”
4. Amhara	9	<1	More perceived benefit from Plumpy'Nut	“I have seen change in appetite after Plumpy'Nut. After he started using Desta, he started to eat less. I saw a change in appetite after Plumpy'Nut but not after Desta.”
5. Amhara	6	2	No perceived benefit	“They have told us to go and get Desta but we didn't go. Even though they told us to collect it, I said to myself, the rest of my kids before him grow up just fine without Desta.”
6. Amhara	18	4	Child refused food with Desta	“At first she ate food with Desta properly, but one day she refused the food and I thought she got tired of it. I also tried to give her after few days but she still refused food with Desta. I tried to give it to her for days and she still hates it. I stopped giving her when there are still four sachets left.”
7. Amhara	18	2	Missed refill appointment, did not know how to obtain more Desta	“I was not in town on the day Desta is distributed, I was away. When I returned I didn't know when the next appointment was, they didn't tell me when it is given or when I should come.”
8. Tigray	7	<1	Child refused food with Desta	“Initially she was taking Desta very well but then she disliked it. She preferred breastfeeding at that time. It could be because of the amount of food I used to mix with Desta. I probably decreased the size of the food I used to mix with the Desta … At the beginning, she was taking it without hesitating but over time she decreased and resisted to eat food mixed with Desta. I am not clear how she could detect it was not good whether it is by taste or smell. For me, I did not sense any smell with it.”
9. Tigray	6	<1	Child has poor overall appetite for foods; child refused food with Desta	“My child has a poor appetite … He is not good with foods other than breastmilk! … But when I started feeding I saw a yellow colour in the and I was astonished by the colour I saw in the food, and I questioned what it could be but I gave to my child and he ate a small amount. But later on the totally refused eating and consequently, I stopped getting Desta from the health post.”
10. Tigray	6	<1	Child has poor overall appetite for foods; child refused food with Desta	“She took it easily, for a while but, later refused to eat, even food without Desta. I tried to change the food which Desta will be added but she totally refused.”
11. Tigray	8	6	Child had diarrhoea and mother scared that giving Desta would continue the diarrhoea	“I stopped giving Desta because my child was sick with diarrhoea repeatedly. The HEW treated my child and counselled me that this is quite normal, it is temporary and the diarrhoea will stop by itself so I should not worry. But I was scared of the diarrhoea so I stopped giving DESTA.”
12. Tigray	8	3	Waiting to restart Desta until child's appetite returns after illness; too busy to obtain refill from HEW	“At that time he was not healthy, he was rejecting any type of food including that with Desta. I took him to the health center get treated and he became well, so because of his illness he stopped taking Desta but only for that specific time. But I will wait some time to continue Desta until his appetite reverses … Desta is useful it does not cause illness, it's my own problem, I failed to bring another Desta because I was so busy doing farming for the last three months.”
13. Tigray	7	6	Perceived Desta as aggravating diarrhoea from existing stomach infection	“I do not think his illness is only because of Desta because even when I stopped Desta his appetite is still low and also he has some diarrhoea though it is not as severe as before. I believe that he has bacteria in his abdomen and this is being aggravated by the introduction of Desta.”
14. Tigray	16	4	Child refused food with Desta; perceived child appetite decreased because of Desta	“One day I tasted it to check why she is refusing it and realized that it has some bitter taste. I realized that it is because of the bitter taste that she is refusing … the HEW taught us that Desta increases the appetite but in my child the reverse happened. She has significantly decreased her appetite once I started feeding Desta. They told us that her stool colour might be changed. I have seen and accepted that it is due to Desta. But when her appetite gets lower and she refuses Desta, I just stopped giving her.”
15. Tigray	8	10	Away from home without appropriate food to mix Desta; too busy to obtain refill from HEW	“Initially I was taking Desta because I had enough time for a refill and I was giving Desta comfortably and I had time, but now I am one of social justice committee and the circumstances that I stay in meeting the whole day, and I took my kid with me and I do not consider to add Desta in food because most of the time the food I took for my child when I have meetings is dry food ‘kita’ and can't get time to add and bring Desta.”
16. Tigray	8	<1	Child refused food with Desta; organoleptic changes to food with Desta; perceived Desta caused diarrhoea and dark stool; perceived child's health diminished; too busy to obtain refill from HEW	“There were many reasons [for stopping Desta]. My child totally refused foods prepared with Desta. Most of the time I dumped the food prepared with Desta because it changed to a yellow colour and the taste become offensive … The other reason was that the child had diarrhoea and at the same time the stool became darker and darker … Totally the health of the child has diminished and I decided to stop feeding because I assumed that the reason for the change in the health of my child was because of Desta. Meanwhile, the last reason that made me stop feeding Desta was because I was busy and I was not able to visit the health post during the appointment time and then I stopped feeding.”

**Table 5:** Positive changes and negative side effects associated with Desta use that mothers reported learning and observing (*n* = 30, including 14 continuing users and 16 noncontinuing users)

	Mothers reported learning about		Mothers reported perceiving in child	
	Continuing user (*n*, yes)	Noncontinuing user (*n*, yes)	Continuing user (*n*, yes)	Noncontinuing user (*n*, yes)
Positive changes				
Improve child's overall health	10	9	8	2
Improve child's intellectual development	7	7	0	0
Increase child's appetite	4	8	6	6
Improve child's growth	6	4	0	0
Improve child gain weight	3	5	5	2
Increase child's strength, “build body”	6	2	6	3
Good for child	3	4	0	0
Help child be more active, give child more energy	5	2	7	1
Improve child's physical development, help child to sit/crawl/walk sooner	2	2	4	1
Make the child happy	1	1	0	1
Decrease anaemia	1	1	0	0
Improve child's overall development	1	1	0	0
Destroy intestinal parasites, “take bad things out of stomach”	0	2	0	1
Other positive changes[Link]			3	1
Side effects				
Dark stool	2	4	4	7
Mild constipation	0	0	0	0
Nausea	0	0	0	2
Diarrhoea	3	4	2	3
Vomiting	0	0	1	4
Child's appetite *decreased*			0	5
Child's health *deteriorated*			0	1

Other positive changes: “Make child normal,” “child became beautiful,” “child can differentiate people,” and “child is alert, knows surroundings.”

At first, she did not take food well, but since I started feeding her Desta her appetite has improved … after I started using Desta she is eating foods well.

Yes, she became strong, she knows her surroundings, she is active. She used to have a stomach ache before, but now she is free from that. She used to get sick, but now that doesn't happen anymore. She is very fine.

When they first gave me Desta I didn't think it was useful, until I noticed a change in her appetite … I felt happy, because she gained weight and her appetite increased.

I was happy when I saw that it makes a child gain weight and build the body. I said to myself that the government must bring this thing, because it is useful.

Four mothers explicitly articulated the role that positive changes played in their continuing to give Desta. The following quote from a mother in Tigray illustrates this idea:

I didn't see any complication and the response was positive, like increasing my child's appetite, I decided to continue and I never missed my appointment so far.

#### 3.2.2 Theme 6. Positive statements about Desta from relatives or neighbours encouraged mothers to maintain the practice of giving it

Just as the influence of others played a role in mothers' decision to try Desta, spontaneous reports of positive comments from others appeared as a theme related to continuing use. This theme emerged not only in the narratives of women who were continuing users (five mothers) but also in noncontinuing users (three mothers). Women discussed how husbands, neighbours, and even older children encouraged and supported them to keep feeding Desta:

We will ask each other whether my child is taking Desta or not? I also ask her whether her baby is taking Desta. Then we will advise [each other] not to discontinue Desta. This is what we say to each other.—Continuing user from Amhara

My husband knows about the advantage of Desta and he has been encouraging to continue feeding Desta.—Continuing user from Tigray

Interviewer: Have you discussed with your husband about him refusing? Caregiver: Yes … he told me to keep trying to feed him whether he took one bite or two bites. He told me to try.—Noncontinuing user from Amhara

#### 3.2.3 Theme 7. Mothers who were informed of potential side effects reported that they were not deterred from continued use of Desta when their children experienced side effects

More than half of the mothers (seven continuing users and 10 noncontinuing users) reported their child had experienced side effects (Table 4). However, the importance of HEW communications about side effects and the benign nature of these side effects emerged as a theme in mothers' discussions. For example,

The health extension workers informed us prior that their faeces might change when children start taking Desta. They advised us not to get worried when their faeces get darken … They just told me that their faeces will get darken. Since I am aware of this, I didn't go for any advice.—Continuing user from Amhara

But I was aware of diarrhoea. The health extension worker told me it is common for a child to have diarrhoea and stool colour change after taking the first doses of Desta. I was not worried about it … They assured me that it can cause such problems, but resolves over time. I then continued to give Desta every third day.—Continuing user from Tigray

A noncontinuing user from Amhara even perceived a change in the child's stool colour to be a good thing, as she interpreted it as a sign Desta was working:

I told you that his stool became darker, other than that there is no change. I thought to myself if his stool is becoming darker it means it is helping him take out bad things from his stomach. I thought it is good.—Noncontinuing user from Amhara

#### 3.2.4 Theme 8. Caregivers spontaneously reported failures of their problem‐solving strategies to overcome challenges in feeding food mixed with Desta

The theme of failure of problem‐solving strategies appeared primarily in the narratives of noncontinuing users. A few of the noncontinuing users expressed their frustration or unhappiness that their attempts did not work. For example,

I tried all my best … since it is very essential for her … I wanted to feed her … but she refused … I tried … but … she refused.—Noncontinuing user from Amhara

Eight noncontinuing users and one continuing user described discussing the problem of their child's refusal of foods mixed with Desta with their HEW. One continuing user received counselling from her HEW on how to better prepare foods with Desta. Six of the eight noncontinuing users also reported receiving advice about what to do in the face of child refusal. However, according to their narratives, the suggestions these six mothers received were primarily to “keep trying” or to “try with different foods.” An idea expressed by three noncontinuing users indicates that there was also a feeling, even among those who described problem‐solving techniques, that nothing could be done if a child refused the food. One noncontinuing user also tied this sentiment to her explanation about why she did not seek help from the HEW:

Interviewer: “So if you felt concerned this much, why didn't you try to make some more efforts? Or take her to [the HEW]?”Caregiver: “No, I didn't take her. What can the HEW do, if she once refused to eat the food?”

#### 3.2.5 Theme 9. The perception of negative effects associated with consuming Desta emerged as a theme for discontinuing its use

The theme of negative effects of Desta was identified in some of the narratives from mothers. Five mothers brought up the problem of loss of appetite and one mother said that her child experienced an overall decrease in health. The following quotes from two noncontinuing users in Tigray illustrate this theme:

The HEW taught us that Desta increases the appetite of the children, but with my child the reverse happened; she has significantly decreased her appetite, once she started eating Desta added to food … when her appetite got lower and refused Desta, I just stopped giving it to her.

Totally the health of the child has diminished and I decided to stop feeding because I assumed that the reason for the change in the health of my child was because of Desta.

### 3.3 Difficulty obtaining a new supply of Desta

The findings in this section were obtained from a general question: “Could you tell me how you collect a new supply of Desta?” By way of background for this question, the programme initially gave mothers a box of Desta containing 30 sachets, together with use and dosing instructions and the Desta child card. The child card included appointment dates for mothers to return to the health post to receive a new box of Desta, which was scheduled 2 months after the date of the current distribution. In the next paragraphs, we present the results from this broad question.

Only half of mothers in the study spontaneously reported that the instructions from their HEW included the information that they should return to the HEW to receive more Desta 2 months after receiving the first box. Six mothers reported that they had returned to their HEW to obtain more Desta 2 months after they received the first box. However, this number reflects the fact that some of the mothers in the sample had not yet reached the end of their initial 2 months at the time of the interview, and some of the noncontinuing users had already decided to stop giving Desta for reasons that had nothing to do with problems in obtaining the next box.

Summarizing the narratives that were elicited with the basic question, we found that several mothers expressed confusion about how to get a refill of Desta or said they had experienced problems. A few mothers did not know when or how to get a refill; several of them thought they could *only* get a refill on their appointment day, without a clear plan of what to do if they missed it. From the narratives, it appears that the appointment card did not play an important role in facilitating refills. Many mothers did not have a card or had lost it, but they thought that they could go back to the health post without it. Some who had an appointment card were illiterate, thus, it was not helpful for them. A few said they did not need the card when they went to the health post because not having it did not keep them from receiving Desta. Some of the expressions of these findings are below:

Interviewer: Why didn't you go when you returned? (from being away when distribution happened) Caregiver: When I returned I didn't know when the next appointment was. They didn't tell me when it is given or when I should come.—Noncontinuing user from Amhara

They didn't tell us if we miss our appointment day that we can take on another day. Since I didn't go on the day of distribution I didn't dare to go and ask … They did not tell me like this (that she could go on a day other than her appointment day) that's why I did not go … Since I was not present on the appointed day I did not bother to go.—Continuing user from Amhara

I do not know when I am going to return back for Desta. They did not tell us the actual date. I did not read it though they have written something over the card.—Continuing user from Tigray

Similarly, a continuing user from Amhara said that she had used her last Desta sachet the week prior to the interview. She said she did not have her refill yet because she missed her appointment day when she was away visiting family. Although she intends to get a refill, she seemed confused as to when she could do this.

Some mothers said they were too busy to obtain a refill of Desta or continue preparing it. The following statements from three noncontinuing users in Tigray illustrate this:

Initially I was taking Desta because I had enough time for a refill and I was giving Desta comfortably and I had time, but now I am one of social justice committee and the circumstances that I stay in meeting the whole day, and I took my kid with me and I do not consider to add Desta in food because most of the time the food I took for my child when I have meetings is dry food “kita” and can't get time to add and bring Desta.

No no, Desta is useful it does not cause illness, it's my own problem, I failed to pick up another Desta because I was so busy doing farming for the last three months.

Meanwhile, the last reason that made me stop feeding Desta was because I was busy and I was not able to visit the health post during the appointment time and then I stopped feeding.

Mothers' knowledge about how to dose Desta is fundamental for obtaining a new supply. The initial offering consisted of one box of Desta, which was supposed to last for 60 days. Although not all mothers spontaneously described the full content of the instructions they received from their HEW, or the written materials provided by the HEW, half of the respondents (eight continuing users and seven noncontinuing users) reported giving Desta to their child every other day (Table 3). In contrast to the mothers who reported correct dosing behaviours, some mothers did not follow the instructions. A continuing user in Tigray reported mixing Desta into portions of food that she gave to both of her young children at each meal every day, for an approximate total of 1.5 sachets daily per child. Another example, from a continuing user in Amhara who gave her child Desta every day, is her statement of her rationale:

We bring Desta and give her on a daily basis. Why do I give her one today and skip tomorrow what reason do I have? So I will give her tomorrow again, but I will not give twice on the same day.

## 4 DISCUSSION

The results presented in this paper were obtained through in‐depth interviews, using a focused ethnographic methodology. The format of the questions and the interviewing method were designed to maximize the opportunities for mothers to articulate their experiences with MNP, their interpretations of its purposes and effectiveness, their management of the problems they encountered, their understanding and interpretations of their interactions with the programme delivery system and its representatives (the HEW), and the challenges and opportunities they experienced in the adherence process. Together with the results of a larger survey (Tumilowicz et al., 2019), which provided the opportunity to conduct a quantitative analysis of the adherence process, the mixed‐method approach that was used to conduct the formative evaluation of the Desta MNP project in Ethiopia is intended to contribute to future nutrition interventions to improve infant and young child nutrition, as well as to provide guidance for the programme in Ethiopia.

A recent review of literature with content related to adherence to MNP provided to children 6–59 months of age found that only 12 of the 35 studies reviewed used qualitative methods and only three of those (Kodish, Rah, Kraemer, Pee, & Gittelsohn, 2011; Loechl et al., 2009; Pelto et al., 2015) were process or formative evaluations that explored mothers' experiences with MNP programmes (Tumilowicz, Schnefke, Neufeld, & Pelto, 2017). Thus, this paper contributes specifically to filling the knowledge gap about mothers' perspectives and experiences with MNP.

Shifting attention to the narrative reports of children's behavioural reactions, the finding that many mothers said their child apparently differentiated between foods prepared with MNP and foods without them is one of our most striking findings. Nutrition professionals have adopted the view that if the food is properly prepared (following the guidelines), infants and young children cannot differentiate food that contains MNP. Our results, as well as a recently published sensory evaluation study of MNP (Sutrisna, Vossenaar, Izwardy, & Tumilowicz, 2017), do not support that contention as a blanket statement about child response. Undoubtedly some of the reported differentiation can be attributed to poor preparation by the mother, and/or by subtle cues from the mother that somehow signal to the child that the food with MNP is different. However, the mothers' narratives suggest that child acceptance is not a foregone conclusion, even when women follow the recommendations and procedures they have been taught.

Among the mothers in our sample who cited rejection of food with Desta as a reason for stopping it, all except for one reported initial periods, even up to 9 months in duration, when the child accepted it. This suggests that one of the causes of child refusals could be related to taste preferences. A strategy of repeated exposure to a new food, combined with supporting caregiver persistence, might increase acceptance of MNP (Moding, 2018; Mura Paroche, Caton, Vereijken, Weenen, & Houston‐Price, 2017). Indeed, repeated feeding was a strategy reported by mothers to overcome initial negative reactions to food mixed with MNP. However, there are also other factors to consider. Several mothers reported child refusal of food with Desta together with general poor appetite and diarrhoea. This suggests that illness and developmental changes are affecting children's acceptance of food with Desta. For example, many children in the second and third year of life enter a “neophobic” phase during which previously liked foods are no longer accepted and the introduction of new foods becomes difficult (Nicklaus, 2009). This inference is supported by survey data analysis, which showed among children whose mothers initiated feeding Desta that children aged 12–17 months and 18–23 months were 32% (*P* < 0.001) and 38% (*P* < 0.001) less likely respectively to have been fed it in the 14 days prior to the interview as compared with children aged 6–11 months—indicating continuing use was more problematic among older children (Tumilowicz et al., 2019). Most studies on the development of taste preferences and caregivers' role in encouraging children to eat less desired foods come from high‐income countries (Mura Paroche et al., 2017). Our study highlights the need for further research on the development of taste preferences and mother–child interactions around food with and without MNP, at different ages and stages of development in resource‐constrained environments. Moreover, it is unwise to assume that all infants and children will readily accept foods with MNP for long periods of time.

With respect to the challenges that mothers faced to overcome child refusals of food with Desta, one is reminded of the earlier insistence of nutrition and health professionals that “breastfeeding is natural, so women should not have problems with it,” and “mothers just need to get on with it” (Shannon, O’donnell, & Skinner, 2007). Fortunately, the development of lactation counselling and lactation counsellors has created an environment in which many women today have access to the help and support their need to sustain breastfeeding (Patel & Patel, 2016). By analogy, the results of this study, including not only the findings concerning reports of negative child reactions but more importantly the fact that those negative reactions lead to cessation of giving MNP, call for attention to developing support systems for women to address negative child reactions to food prepared with MNP and to instituting them as an integral part of programs to improve child nutrition through MNP.

The reported experiences with side effects are, in our view, another important finding from the study. It is very encouraging to see that when health workers advise mothers about side effects, to the level of being very explicit about potential observable symptoms, it moderates the mothers' reactions when those side effects occur. It was a wise decision on the part of the programme to include these in the BCC messages for mothers.

It is also encouraging to note that the programme provided nuanced information to mothers, in the form of suggesting there are signs and symptoms “your child *may* experience,” laying the groundwork for differential reactions, as contrasted with a blanket statement about reactions. This tacit message about individual differences accords with mothers' experiences in other domains of health and development. It is, in that sense, a “meta‐message” that can be simultaneously reassuring for women and open future lines of dialogue between health providers and mothers.

Regarding mothers' spontaneous reports of side effects and the finding that only some respondents commented on them, we note that this differential reporting may reflect actual differences in observable symptoms or differences in mothers' attention to and sensitivity to their children's responses and behaviours. Without observational studies, this question, clearly, cannot be answered.

Concerning the problems women reported about getting refills, the narratives provide evidence that confusion about what to do was a significant barrier to the final step in adherence—continued use. We note that the set of messages HEW were given to convey to mothers about this aspect of the programme was inadequate, and mothers' confusions about the process may stem from that programme‐originated problem. This interpretation gains plausibility because the messages related to dosing, which were well developed, were understood and followed by half of the women we interviewed. At the same time, we note the appearance of other “refill barriers,” particularly constraints on women's time to acquire refills, and absence from their home base, as factors that also affected continuing use of MNP.

Finally, it is important to point out that all the data presented in the paper represent the perspectives of mothers who tried Desta. The paper does not address the issue of factors that prevented mothers from trying Desta after being presented with the product or learning about it, or who did not learn about it, even though they lived in the project catchment areas. On the basis of the neutral or positive insights about the mother's experience with programme delivery and the overwhelmingly positive response to learning about the product itself and its benefits for children, we surmise that the main limiting factor for not trying Desta was not encountering the programme delivery, the BCC activities, or the product at all. This inference is supported by the survey results, which showed that among mothers who received Desta, 98% tried it (Tumilowicz et al., 2019). Thus, we conclude that ensuring mothers learn and receive Desta is critical because they are very likely to try it initially if they are exposed to the product and learn about its use and benefits.

## ACKNOWLEDGEMENTS

We acknowledge the cooperation and support extended by officials from the Federal Ministry of Health (FMOH) and regional and district health offices in Ethiopia. We also acknowledge Concern Worldwide for supporting programme implementation. We are indebted to the data collectors from Nutrition Unit, School of Public Health, Addis Ababa University for making concerted efforts to ensure the proper gathering and organizing of relevant data required for this study. We acknowledge the assistance of Marieke Vossenaar in preparing the manuscript for publication.

## CONFLICTS OF INTEREST

The authors declare that they have no conflicts of interest.

## CONTRIBUTIONS

AT, GHP, CHS, SH and LMN designed the study; SH and MH conducted data collection; CHS, GHP, SH, AT, WG, MH and HYW conducted data analysis and interpretation of results. GHP, AT and CHS wrote the first draft of the manuscript; all authors read, revised and approved the final version submitted for publication.
